# New Onset Refractory Status Epilepticus as an Unusual Presentation of a Suspected Organophosphate Poisoning

**DOI:** 10.1155/2014/676358

**Published:** 2014-12-17

**Authors:** Shahan Waheed, Amber Sabeen, Nadeem Ullah Khan

**Affiliations:** ^1^Department of Emergency Medicine, Aga Khan University Hospital, Stadium Road, Karachi 74800, Pakistan; ^2^Department of Internal Medicine, Aga Khan University Hospital, Stadium Road, Karachi 74800, Pakistan

## Abstract

New onset refractory status epilepticus (NORSE) is a new entity in medical literature. It has different infectious and noninfectious etiologies showing a devastating impact onto the clinical outcome of patients. Therapy with anaesthetic and antiepileptic agents often fails to improve the condition, unless the primary cause is rectified. Here is presented the case of a young female with a history of depression who after a recent bereavement came to the Emergency Department of Aga Khan University Hospital with complaints of drowsiness that lasted for few hours. Though she had no history of organophosphate poisoning, her physical examination and further investigations were suggestive of the diagnosis. During her hospital stay, she developed refractory status epilepticus. Her seizures did not respond to standard antiepileptic and intravenous anesthetic agents and subsided only after intravenous infusion of atropine for a few days. Organophosphate poisoning is a very common presentation in the developing world and the associated status epilepticus poses a devastating problem for emergency physicians. In patients with suspected organophosphate poisoning with favoring clinical exam findings, the continuation of atropine intravenous infusion can be a safe option to abate seizures.

## 1. Introduction

Organophosphates are some of the most common agents of poisoning that account for approximately 60% of all poisoning cases in Pakistan [1]. It is considered that the primary mechanism of action of these compounds is the irreversible inhibition of acetylcholinesterase (AChE), which results in the accumulation of acetylcholine at the nerve synapses and the stimulation of seizure activity (convulsions). This, when coupled with the existence of excitatory neurotransmitters, activates the NMDA receptors in the brain, eventually leading to status epilepticus [2].

New onset refractory status epilepticus (NORSE) is a relatively new addition in the medical literature and is used to describe a condition where previously healthy adults now present with de novo refractory status epilepticus [3]. An infectious insult, especially viral encephalitis, is regarded as a harbinger for its onset but evidence is still lacking to confirm this [4].

In organophosphate poisoning, status epilepticus is associated with different chemical warfare agents like sarin, sobun, and tabun. The toxicity of these agents is focused on the central and peripheral nervous systems [5]. Here is reported the case of a patient presenting with NORSE and organophosphate poisoning which was suspected on the basis of clinical features and low serum acetylcholinesterase levels.

## 2. Case Presentation

A 27-year-old woman, recently bereaved due to the death of her 6-month-old daughter, came to the emergency with the sudden onset of drowsiness that lasted for 4 hours. She had no history of poisoning or drug overdose. On her physical examination, a clear observation of pinpoint pupils, bradycardia, bronchorrhea, and the drooling of saliva raised strong suspicions of organophosphate poisoning, which is usually one of the most common types of poisoning in developing countries.

Her treatment started with the administration of intravenous atropine and pralidoxime based on her physical examination findings. A few hours later she developed seizures for which she was administered intravenous benzodiazepine. Despite treatment, the seizures continued. She was given multiple antiepileptic drugs, none of which controlled the seizures. She was then intubated due to her condition of having refractory status epilepticus and the electrolytes were normal. She was put under 24-hour EEG monitoring, which showed continuous seizure activity. CSF studies along with brain imaging to point towards any infectious or ischemic etiology for her condition were found to be negative. Meanwhile she remained on intravenous atropine and pralidoxime. Her seizures were controlled after 3 days of treatment. When intravenous administration of atropine and pralidoxime was stopped, the seizures recommenced within a few hours. Her intravenous anesthetic dose was increased and she was put on a combination of five antiepileptic medications. Based on her examination findings and clinical parameters, the treatment combo of intravenous atropine and pralidoxime was restarted. She continued to have seizures for 6 days. Finally her seizures were controlled after 10 days via application of intensive intravenous anesthetic agents, antiepileptic medications, and atropine infusion. The application of antiepileptic medications, atropine, and intravenous anesthetic agents was gradually tapered. The patient was extubated after a few days and remained well afterwards. She was discharged home on prescription of oral levetiracetam and phenytoin with neurology follow-ups.

## 3. Discussion

This case highlights a very serious and lethal manifestation of organophosphate poisoning, which was NORSE caused by a possible household agent. We were unable to identify the substance the patient had ingested, and she had no history of fumigation within her house. Data from the National Poison Control Centre, Karachi, Pakistan, reported 1999 cases of organophosphate poisoning with CNS manifestations including only altered consciousness and seizures, but not NORSE [1]. Review articles on neurotoxic effect of organophosphate poisoning also failed to mention such a severe CNS presentation [6]. This case emphasizes the need for having stringent regulations regarding the use of pesticides in Pakistan. According to WHO, extremely hazardous (class 1a) and highly hazardous (class 1b) pesticides [7] should not be used in households; they should be available only to licensed personnel and should bear a label of either “poison” or “toxic” to indicate a high degree of hazard. Failure in complying with these regulations leads to higher mortality in developing countries. Ban on sale of these products may improve poisoning related deaths in developing country like Pakistan [8].

This case also highlights the importance of recognizing toxidromes to reach a diagnosis, as most of the time the history of toxic ingestion is not available. The patient was in cholinergic crisis, as recognized solely on clinical manifestation. This cholinergic crisis could also have been due to some plant poisoning through alkaloid compounds such as solanine that inhibit cholinesterase enzyme similar to an organophosphate compound [9]. The specific management of a severe cholinergic crisis due to the cholinesterase inhibition is the intravenous administration of atropine/pralidoxime as antidote and supportive care with fluids and electrolytes. In this particular case, the patient was managed on the basis of clinical findings alone, and later on diagnosis was confirmed through serum acetylcholinesterase levels. As testing for RBC cholinesterase levels is not available in Aga Khan University Hospital, serum cholinesterase levels were considered in diagnosis. This case also suggests that poisonings should always be considered in differential diagnosis of any new symptomatology, because specific treatment would affect the outcome as described in this case. The risk of poisoning/self-harm was suspected as she had gone through a recent bereavement and as per her husband she had been depressed after the death of her child who died as a result of an accident and she was on antidepressants. She was a housewife and was living with 3 children. Also she has both clinical features and low cholinesterase levels.

Organophosphate is the most common cause of poisoning in Pakistan because it is cheap and easily available over the counter and has no regulatory control [10].

We had been doing serial blood cholinesterase levels as it is the test that is available in Pakistan and in our institution. The RBC cholinesterase level although more sensitive is not available in our institution. Both atropine and pralidoxime infusions are continued until the resolution of symptoms and signs. The atropine infusion was continued at 2 mg per hour. The patient was followed clinically both for resolution of cholinergic symptoms and for cholinesterase level that showed a rise. Serial cholinesterase level not only has a good sensitivity but also has a prognostic value in terms of outcome in organophosphate poisoning [11, 12].

The average duration of NORSE as reported by Costello et al. is believed to be 36 days (6–68 days) [13], and a case from Verma et al. had the duration of 12 days with herpes simplex encephalitis as the putative agent for the event. The case discussed in this paper/report had a probable toxic insult which lasted for 10 days after the continuation of the atropine infusion. Also, patients who were presented in the past with NORSE had some common clinical features which include female sex, young age at presentation, no prior history of any neurological disorder or seizures, neuroimaging, and CSF abnormalities, all of which were consistent in our patient. The association of the status epilepticus with chemical warfare agents is shown in the literature. The above case presentation points towards thinking of whether any substance was used in the household or there was any other cause for these clinical features and low serum acetyl cholinesterase levels.

In the treatment of the patient discussed here, multiple antiepileptic drugs were tried which were unsuccessful in controlling seizures. One recent study reported the efficacy of sec-butyl-propylacetamide (SPD), a one-carbon homolog of valnoctamide (VCD), which is a central nervous system- (CNS-) active amide derivative of valproic acid (VPA), to be effective in diazepam resistant cases of status epilepticus in organophosphate poisoning cases. This drug is currently in phase II of clinical trials.

## 4. Why Should an Emergency Physician Be Aware of It?

New onset refractory status epilepticus in patients with clinical features suggesting organophosphate poisoning is a rare entity. In patients with suspected organophosphate poisoning with favoring clinical exam findings, the continuation of atropine intravenous infusion can be a safe option to abate seizures.
